# Two pediatric oncologic cases of hypereosinophilic syndrome and review of the literature

**DOI:** 10.1002/cnr2.1710

**Published:** 2022-10-14

**Authors:** Julie Voeller, Thomas DeNapoli, Timothy C. Griffin

**Affiliations:** ^1^ Department of Hematology Oncology The Children's Hospital of San Antonio, Baylor College of Medicine San Antonio Texas USA; ^2^ Department of Pathology The Children's Hospital of San Antonio, CHRISTUS Health San Antonio Texas USA

**Keywords:** leukemia, molecular diagnosis, pediatric cancer

## Abstract

**Background:**

Persistent peripheral blood hypereosinophilia may cause tissue damage, leading to hypereosinophilic syndrome (HES) with end‐organ dysfunction. Here we discuss two unique pediatric cases of primary hypereosinophilic syndrome with oncologic etiologies to highlight the importance of early recognition, workup and treatment of HES.

**Case 1:**

A previously healthy 7‐year‐old male presented with acute myocardial infarction and transient ischemic attack and found to have significant hyperleukocytosis with a total white blood count of 131 000 and hypereosinophilia with an absolute eosinophil count of 99 560. He was ultimately diagnosed with precursor B‐cell acute lymphoblastic leukemia with immunoglobulin heavy chain gene rearrangement. He completed standard treatment without significant complications and remains in remission at about 2 years off therapy. He is in overall good health and has normal cardiac function.

**Case 2:**

A 13‐year‐old female was referred for iron deficiency and reported a history of severe anxiety, shortness of breath and anorexia. She had experienced fatigue and dizziness associated with frequent panic attacks and shortness of breath with strenuous activity since the age of five. Serial laboratory investigations revealed persistent hypereosinophilia (AEC 4000‐6000/μl). Additional workup revealed elevated vitamin B12 (>2000 pg/ml; normal range: 243–894) and tryptase (16.4 ng/ml; normal range: ≤10.9). The FIP1L1‐PDGFRA gene fusion was detected by fluorescence in situ hybridization (FISH) on peripheral blood, diagnostic for myeloid/lymphoid neoplasm with eosinophilia. Evaluation for end‐organ damage associated with persistent hypereosinophilia included an echocardiogram which revealed severe restrictive cardiomyopathy with pulmonary hypertension. Monotherapy with imatinib was initiated, after which she achieved a rapid hematologic response and remains in molecular remission, though she continues to have persistent asymptomatic severe pulmonary hypertension in the setting of severe diastolic dysfunction.

**Conclusion:**

Persistent hyperosinophilia can be a silent cause of significant and often irreversible tissue damage and should therefore always prompt workup for both primary and secondary causes.

## 1 INTRODUCTION

Hypereosinophilia is defined as an absolute eosinophil count (AEC) exceeding 1500 eosinophils/μl in the peripheral blood on two examinations separated in time by at least 1 month and/or pathologic confirmation of tissue hypereosinophilia. Persistent hypereosinophilia may lead to hypereosinophilic syndrome (HES) with end‐organ dysfunction.^1^ After secondary causes are excluded (Table 1), workup for a primary or clonal eosinophilia should begin promptly. In these current cases, we present two pediatric oncologic cases of hypereosinophilia to illustrate the clinical spectrum of HES.

**TABLE 1:** Secondary causes of peripheral eosinophilia

Category	Examples
Atopic disorders	Asthma, allergic rhinitis, atopic dermatitis, allergic bronchopulmonary aspergillosis
Gastrointestinal disorders	Eosinophilic gastroenteritis, celiac disease
Infectious disorders	Helminthic or parasitic infection, fungal infection (coccidioidomycosis, cryptococcus)
Autoimmune disorders	Churg‐Strauss syndrome, sarcoidosis
Malignancies	Hodgkin and non‐Hodgkin lymphoma, acute lymphoblastic leukemia
Immunologic disorders	Hyper‐IgE (Job) syndrome, Wiskott‐Aldrich syndrome
Drug reaction	Drug reaction with eosinophilia and systemic symptoms (DRESS), IL‐2 therapy
Other	Adrenal insufficiency

### 1.1 Case 1

A previously healthy 7‐year‐old male presented to The Children's Hospital of San Antonio (CHOSA) in January 2017 with acute onset headache, blurry vision, and left arm and chest pain. Review of systems was significant for fever, decreased appetite and a 12‐pound weight loss over the last month. Laboratory investigations revealed a total white blood count (WBC) of 131 000/μl (normal range: 4500‐12 500/μl) with 76% eosinophils (AEC 99 560/μl; normal range: 200–600/μl), a hemoglobin of 11.4 g/dl (normal range: 11.5–15.5/dl), and a platelet count of 98 × 10^9^/L. A brain MRI showed innumerable acute ischemic infarcts. An electrocardiogram revealed non‐ST‐elevation myocardial ischemia (NSTEMI).

**FIGURE 1:**
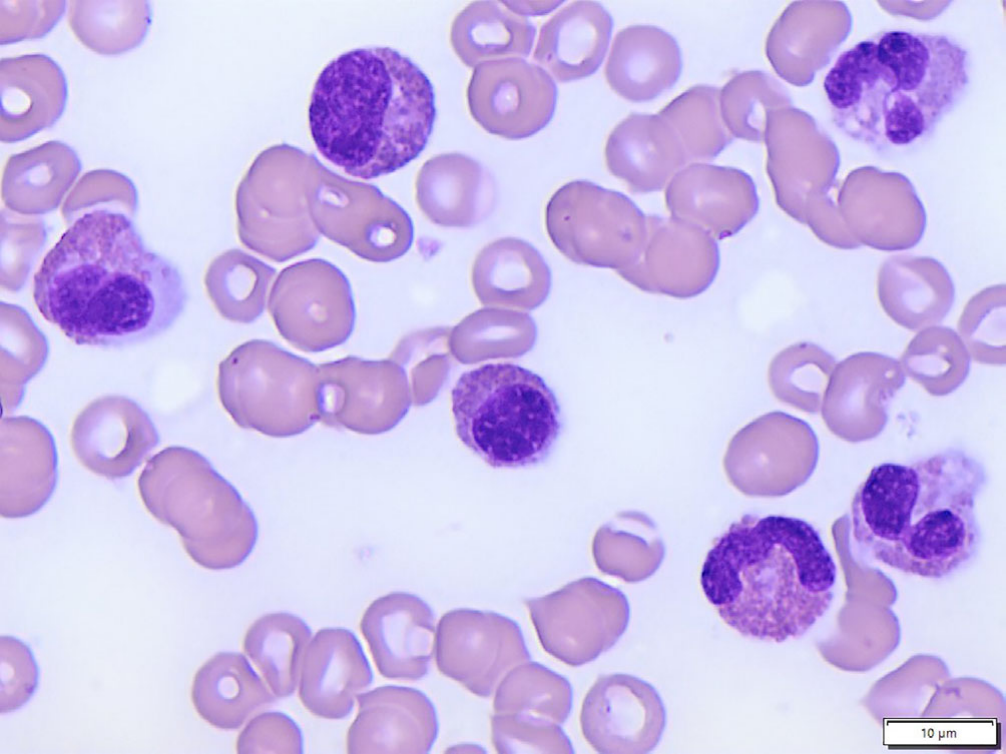
Wright‐Giemsa‐stained bone marrow aspiration slide showing bone marrow eosinophilia, including some showing hypogranulation (100× oil)

Initial bone marrow biopsy revealed marked bone marrow hypereosinophilia (Figure 1) but <5% morphologic blasts. Concurrent flow cytometric evaluation, however, showed 0.8% CD10/CD19‐positive cells. Additional cytogenetic, fluorescence in situ hybridization (FISH) and molecular studies were negative, including PDGFR, PDGFRB, and FGFR1 probe sets. He was administered high‐dose methylprednisolone (30 mg/kg/day for 5 days) and hydroxyurea (500 mg/day) for treatment of idiopathic HES.^2^, ^3^ Due to his critically‐ill status and persistent hypereosinophilia, the patient was continued on prednisone 10 mg/kg/day, hydroxyurea was increased up to 900 mg/day, and imatinib 300 mg/day was initiated.

**FIGURE 2:**
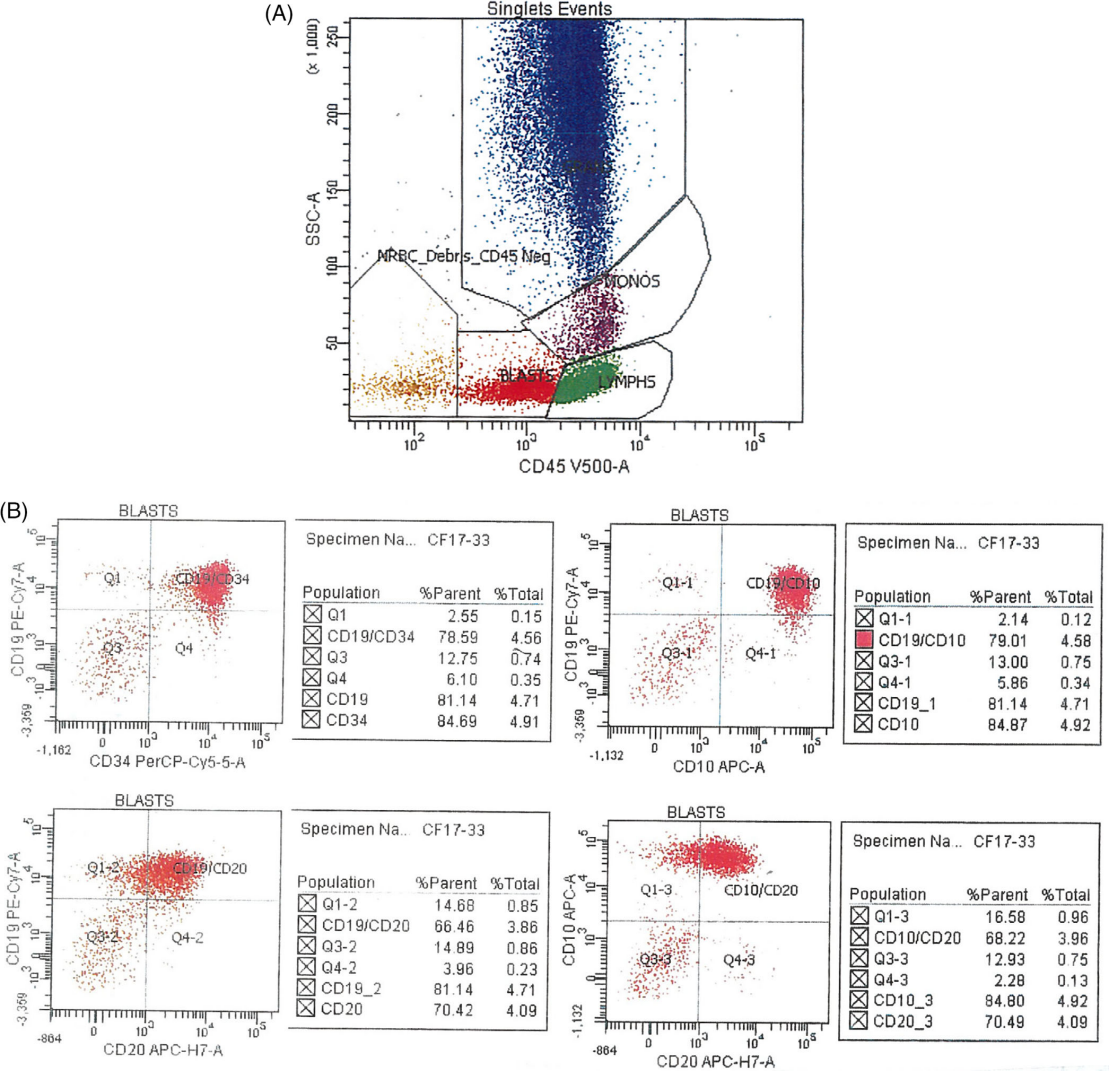
(A) Flow cytometric plot showing 5.5% total cells in blast gate (red arrow). (B) Flow cytometric profile showing CD34, CD19, CD10, CD20 (variable), HLA‐DR, CD58 (moderate), CD123 (moderate), cyCD79, and TDT positivity in the atypical cell population

After 2 weeks, his hyperleukocytosis and hypereosinophilia improved (WBC 4900/μl and AEC 540 μ/L). Repeat bone marrow studies were performed and flow cytometry now showed 5.5% precursor B‐cells with an abnormal phenotype showing CD34, CD19, CD10, CD20 (variable), HLA‐DR, CD58 (moderate), CD123 (moderate), cyCD79, and TdT positivity (Figure 2A,B). FISH analysis was now abnormal showing an immunoglobulin heavy chain (IgH) rearrangement. These findings were consistent with a diagnosis of precursor B‐cell acute lymphoblastic leukemia (ALL) with IgH gene rearrangement. He completed standard treatment without significant complications and remains in remission at about 2 years off therapy. He is in overall good health and has normal cardiac function.

### 1.2 Case 2

A 13‐year‐old female was referred to CHOSA in August 2020 for iron deficiency and reported a history of severe anxiety, shortness of breath and anorexia. She endorsed fatigue and dizziness associated with frequent panic attacks and shortness of breath with strenuous activity since the age of 5. Review of symptoms was otherwise negative. Serial laboratory investigations revealed persistent hypereosinophilia (AEC 4000‐6000/μl). Additional workup revealed elevated vitamin B12 (>2000 pg/ml; normal range: 243–894) and tryptase (16.4 ng/ml; normal range: ≤10.9). A parasitic evaluation was negative and her IgE level was normal. She denied any recent new medication use. The FIP1L1‐PDGFRA gene fusion was detected by FISH on peripheral blood, diagnostic for myeloid/lymphoid neoplasm with eosinophilia (MLN‐eo). Evaluation for end‐organ damage associated with persistent hypereosinophilia included an echocardiogram which revealed severe restrictive cardiomyopathy with pulmonary hypertension.

Treatment with imatinib 100 mg daily was initiated in conjunction with steroids to prevent cardiogenic shock.^4^, ^5^, ^6^ She tolerated treatment without complications and had a rapid hematologic response with resolution of peripheral hypereosinophilia and cytopenias and normalization of her serum tryptase and vitamin B12 levels within a month. She attained molecular remission by 3 months and remains in remission after nearly a year on continued monotherapy with imatinib. During this time, the patient reported improved energy and gained 10 pounds (BMI increased from 1st to 10th percentile). While she remains asymptomatic from a cardiovascular standpoint, she continues to have persistent severe pulmonary hypertension in the setting of severe diastolic dysfunction.

## 2 DISCUSSION

Persistent hypereosinophilia can cause significant tissue damage leading to life‐threatening end‐organ damage, therefore an AEC exceeding 1500 eosinophils/μl on at least two separate occasions at least 1 month apart should immediately prompt additional workup. Here we present two unique oncologic cases of hypereosinophilia, which is a rare entity in pediatrics.

In the first case, the onset of hypereosinophilia was likely subacute, leading to non‐ST‐elevation myocardial infarction (NSTEMI) and symptomatic ischemic infarcts. Almost any leukemia or lymphoma can present with eosinophilia; in children, these cases most commonly present as precursor B‐cell acute lymphoblastic leukemia with *t*(5;14) translocation, which causes fusion of the immunoglobulin heavy chain (IgH) gene to the *IL3* gene promoter. Overproduction of interleukin‐3 drives peripheral hypereosinophilia. As was for the patient in Case 1, most pediatric patients who can tolerate standard intensive induction chemotherapy to treat the underlying disease achieve durable remissions.^7^

In the second case, the onset of hypereosinophilia was likely chronic and present for at least a year prior to initiation of treatment with imatinib. Tissue damage from chronic hypereosinophilia led to significant cardiopulmonary dysfunction. Elevated serum tryptase levels (greater than 11.5 ng/ml) may be a sensitive marker of a MLN‐eo secondary to FIP1L1‐PDGFRA fusion that is characterized by tissue fibrosis and poor prognosis if left untreated.^7^

MLN‐eo with FIP1L1‐PDGFRA rearrangement is rare, with less than 200 reported total cases and only nine previously reported pediatric cases.^8^, ^9^ Patients are responsive to treatment with imatinib and have a 10‐year overall survival of 86%.^4^, ^5^ Despite symptomatic improvement, it is currently unknown if imatinib can reverse eosinophil‐related organ damage.^2^ While treatment‐free remission longer than 3 years has been observed in 30%–40% of patients with FIP1L1‐PDGFRA‐positive MLN‐eo, discontinuation of imatinib should generally be undertaken in the context of clinical trials or registries.^10^, ^11^ Acquired T674I or D842V substitutions in the PDGFRA kinase domain confers resistance to imatinib^12^, ^13^ and may be responsive to treatment with other tyrosine kinase inhibitors.^5^ Refractory cases are typically considered for allogeneic hematopoietic cell transplant.

## 3 CONCLUSION

In summary, persistent hyperosinophilia can be a silent cause of significant and often irreversible tissue damage and should therefore always prompt workup for both primary and secondary causes. While rare, the diagnosis of MLN‐eo can be made rapidly by FISH on peripheral blood for the FIP1L1‐PDGFRA gene fusion and should be performed early in the workup for HES.^4^

## AUTHOR CONTRIBUTIONS

**Julie Voeller:** Conceptualization (equal); writing – original draft (lead); writing – review and editing (equal). **Thomas DeNapoli:** Writing – review and editing (equal). **Timothy C. Griffin:** Conceptualization (equal); writing – review and editing (equal).

## CONFLICT OF INTEREST

The authors have stated explicitly that there are no conflicts of interest in connection with this article.

## ETHICS STATEMENT

Written informed consent was provided by each of the patient's legal guardian.

## Data Availability

Data sharing is not applicable to this article as no new data were created or analyzed in this study.
